# Ocular Gene Therapy with Adeno-associated Virus Vectors: Current Outlook for Patients and Researchers

**DOI:** 10.18502/jovr.v15i3.7457

**Published:** 2020-07-29

**Authors:** Geoffrey A. Casey, Kimberly M. Papp, Ian M. MacDonald

**Affiliations:** ^1^Department of Medical Genetics, Faculty of Medicine and Dentistry, University of Alberta, Canada; ^2^Faculty of Science, University of Alberta, Canada; ^3^Department of Ophthalmology, Faculty of Medicine and Dentistry, University of Alberta, Canada

## Abstract

In this “Perspective”, we discuss ocular gene therapy – the patient's perspective, the various strategies of gene replacement and gene editing, the place of adeno-associated virus vectors, routes of delivery to the eye and the remaining question - “why does immunity continue to limit efficacy?” Through the coordinated efforts of patients, researchers, granting agencies and industry, and after many years of pre-clinical studies, biochemical, cellular, and animal models, we are seeing clinical trials emerge for many previously untreatable heritable ocular disorders. The pathway to therapies has been led by the successful treatment of the RPE65 form of Leber congenital amaurosis with LUXTURNA${}^{\mathrm{TM}}$. In some cases, immune reactions to the vectors continue to occur, limiting efficacy. The underlying mechanisms of inflammation require further study, and new vectors need to be designed that limit the triggers of immunity. Researchers studying ocular gene therapies and clinicians enrolling patients in clinical trials must recognize the current limitations of these therapies to properly manage expectations and avoid disappointment, but we believe that gene therapies are well on their way to successful, widespread utilization to treat heritable ocular disorders.

##  INTRODUCTION

Patients with hereditary retinal disorders are keenly interested in gene therapy. Many recent media presentations feature patients who have experienced apparent benefit from gene therapy treatments, increasing public awareness of gene therapy. It is true that gene therapy may conceptually be an ideal method to treat heritable eye diseases before irreparable damage has occurred to the eye, but it is crucially important to manage patient perspectives around their participation in clinical trials. The leading successful example of ocular gene therapy is LUXTURNA${}^{\mathrm{TM}}$ (voretigene neparvovec-rzyl), an adeno-associated virus (AAV) vector treatment for Leber congenital amaurosis. LUXTURNA${}^{\mathrm{TM}}$ is now approved as a treatment in the USA and the European Union. Unfortunately, many patients believe that enrollment in a clinical trial means “early access” to a treatment or cure, when the trials have not yet reported fully their outcomes. As an example, some patients do not fully understand that LUXTURNA${}^{\mathrm{TM}}$ is designed to treat a single genetic disorder caused by biallelic pathogenic variants in the *RPE65* gene. Patients with a progressive heritable eye disease are a distinctly vulnerable population. They may have incomplete knowledge of their condition and partially formed opinions on risks and benefits of experiments such as gene therapy. They carry with them the hope that a trial may lead to treatment but may also conflate the trial with finding a cure. After the patient passes the screening and consent process, they may face significant personal challenges of a rigorous and frequent follow-up schedule. They (or their families) may not be completely prepared for the possibility of harm or the systemic consequences of high-dose immunosuppression. According to the partner of one patient in the choroideremia clinical trial in Alberta, “[The prednisone] was the hardest part for me. I was angry and upset and after my husband had to start a second round of it. I was kind of thinking, `was this the right thing to do?'^[1]^ Careful consideration of the patient's experience throughout the clinical trial should be considered in the planning stages to avoid miscommunication and psychological harm.

### Gene augmentation vs gene editing

Gene therapies typically employ a gene augmentation or gene editing strategy. In the case of gene editing, the entities introduced into patient cells (for example, a CRISPR/Cas9 system) are designed to correct the mutation in the endogenous copy (or copies) of the gene. In augmentation gene therapy, the mutated/non-functional copies of the gene are ignored, and patient cells are supplemented with a functional copy of the gene.^[2]^ This strategy depends upon sufficiently healthy residual cells being present that can be “rescued” by the therapy. If photoreceptors have already been lost, then alternate approaches must be utilized. In a gene therapy technique called “optogenetics”, non-photoreceptor cells are made photosensitive by expressing channel proteins. A clinical trial of the optogenetic approach is currently recruiting patients with retinitis pigmentosa regardless of the genetic etiology of their condition (ClinicalTrials.gov NCT03326336).

Clinical trials of ocular gene therapies to treat several monogenic disorders (choroideremia, X-linked retinoschisis, X-linked retinitis pigmentosa, achromatopsia, and Leber hereditary optic neuropathy) are underway across North America. AAV is a common choice as a vector for gene delivery. Despite initial hope that AAV vectors would be minimally immunogenic, preliminary results are available and adverse events have been reported in response to the vectors in some cases.

### Routes of vector administration

The route of administration for an ocular gene therapy vector depends on the target cells and the tropism of the vector itself, but will generally fall into one of two categories: intravitreal injection or subretinal injection.^[3]^ Wild-type AAV vector capsids do not penetrate deeply into the retina when administered intravitreally, but the ability of novel AAV capsids (produced via directed evolution) to transduce the posterior cells of the retina is under investigation.^[4,5]^ An additional barrier to successful transduction of any retinal cells via intravitreal injection is the humoral immune system; intravitreal injection of non-human primates with both the AAV2 and AAV8 serotypes was found to stimulate production of neutralizing antibodies in subjects with and without pre-existing immunity to the serotype.^[3,6]^ Conversely, subretinal injection directly exposes the posterior cells of the retina to the vector and evades humoral immune surveillance; however, the retinal detachment required to create space for vector administration triggers microglial activation and photoreceptor death.^[7]^

### The remaining question of immune response

The natural triggers of immunity from the viral vectors remain problematic in experimental therapies.^[8]^ In our center, in an investigator-sponsored (Phase 1) trial of a subretinally injected AAV gene therapy for choroideremia, one subject experienced a serious loss of vision.^[9]^ Unfortunately, the disease progressed without apparent benefit in all patients. We now question why this serious adverse event occurred? Could it have been prevented by an alternative vector design? Why was steroid (given perioperatively) not sufficient to manage inflammation? In our trial, all subjects were treated with high-dose oral steroid (prednisone 1 mg/kg/day 3 days prior to surgery, continuing for 21 days). The steroid was given as a prophylactic to suppress the potential risk to the eye of the subretinal injection. Steroid has both local and systemic anti-inflammatory and immunosuppressive effects; briefly, prednisone inhibits neutrophil migration.^[10]^ It decreases mononuclear phagocyte chemotaxis and the production of interleukins and TNF-α. It causes the redistribution of CD4+ and CD8+ T-lymphocytes, and inhibits T-lymphocyte activation, proliferation, and lymphokine production. At high doses, it inhibits immunoglobulin production by B-lymphocytes.

While prophylactic steroids may aid in regulating players of adaptive immunity such as T-lymphocytes and B-lymphocytes, these cells would not occupy retinal tissue unless a severe breach had occurred in the blood–retinal barrier. For the purposes of retinal gene therapy, local innate immune factors are the primary influencers of an inflammatory response and are of therapeutic importance. To illustrate, our choroideremia gene therapy trial utilized a subretinal injection procedure to introduce an AAV vector into the retina. Retinal detachment induces local TNF-α secretion which in turn may promote autophagy of photoreceptors by resident microglia.^[11]^ Even without considering the immunological effects of the viral vector or the diseased microenvironment of the patients' retinas, the gene therapy procedure itself calls for the incorporation of a local immune regulator to prevent such adverse events.

Both a diseased microenvironment and retinal detachment from the AAV injection “prime” the local immune state to recognize and respond to viral vectors more effectively. AAV viruses are DNA-based and are thus recognized by toll-like receptor 9 (TLR9). TLR9 stimulation in retinal pigment epithelial cells (RPE) by CpG-DNA induces secretion of the pro-inflammatory cytokine IL-8, which represents the initiation of an inflammatory cascade.^[12]^ Preventing this TLR9-initiated inflammatory cascade is essential when designing an immunity regulator in AAV-based retinal gene therapy. Our team is investigating a modified AAV vector that blocks the dimerization and immune signaling of TLR9. Should this vector be effective in preventing RPE from releasing pro-inflammatory cytokines in response to AAV treatment, we will move from cell culture models to animal models to confirm safety of the vector before attempting a second choroideremia gene therapy clinical trial.

Experimental vector gene therapies for hereditary ocular disorders will continue to improve, gain popular exposure, and march toward regulatory approval. A better understanding of the impacts of innate immunity in the retina will improve the safety profile of these therapies and improve the probability of positive outcomes for patients.

##  Financial Support and Sponsorship

Alberta Innovates Health Solutions, Canadian Institutes of Health Research, Fighting Blindness Canada.

##  Conflicts of Interest

There are no conflicts of interest.
